# No-Reference Quality Assessment of Stereoscopic Video Based on Temporal Adaptive Model for Improved Visual Communication

**DOI:** 10.3390/s22218084

**Published:** 2022-10-22

**Authors:** Fenghao Gu, Zhichao Zhang

**Affiliations:** 1 School of Art and Design, Changzhou University, Changzhou 213164, China; 2 College of Electrical Engineering, North China University of Science and Technology, Qinhuangdao 066008, China

**Keywords:** stereoscopic video quality assessment, temporal adaptive module, local and global

## Abstract

An objective stereo video quality assessment (SVQA) strives to be consistent with human visual perception while ensuring a low time and labor cost of evaluation. The temporal–spatial characteristics of video make the data processing volume of quality evaluation surge, making an SVQA more challenging. Aiming at the effect of distortion on the stereoscopic temporal domain, a stereo video quality assessment method based on the temporal–spatial relation is proposed in this paper. Specifically, a temporal adaptive model (TAM) for a video is established to describe the space–time domain of the video from both local and global levels. This model can be easily embedded into any 2D CNN backbone network. Compared with the improved model based on 3D CNN, this model has obvious advantages in operating efficiency. Experimental results on NAMA3DS1-COSPAD1 database, WaterlooIVC 3D Video Phase I database, QI-SVQA database and SIAT depth quality database show that the model has excellent performance.

## 1. Introduction

With the gradual maturity of stereoscopic display technology, video has moved from plane to stereoscopic, and stereoscopic multimedia has entered the daily life of consumers [1]. According to the imaging principle, stereoscopic display technology can be divided into three types, including binocular 3D display, true 3D display and holographic display. At present, polarization 3D projection display technology, namely binocular 3D display technology, is widely used in real scenes such as stereoscopic film projection. In principle, most 3D videos are collected from two groups of videos with slightly different horizontal angles at the source end and transmitted to the audience’s left eye and right eye, respectively, so as to generate 3D in the visual system of audiences. Stereo display has a wide range of related research fields, including stereo image acquisition, stereo positioning, stereo view matching and 3D information reconstruction. The development of these fields provides important support for the deployment of stereo-image/video-related technologies in reality.

Stereoscopic display technology not only enables the audience to enjoy the impact and immersion brought by vision, but also puts forward higher requirements for content and picture quality. At present, 3D video is mainly PGC. In the process of 3D image acquisition, professionals control the quality of video shooting professionally and strictly [2]. However, due to the limitations of hardware equipment and technical level, video has varying degrees of distortion in storage, transmission, display and other links, which makes the viewing experience of users decline. For 3D video, a low-quality content presentation can easily destroy the stereoscopic sense of human eyes, and even cause physiological discomfort to users [3]. Therefore, effective stereo video quality assessment methods are needed to control its quality. In terms of stereo video quality assessment, domestic and foreign scholars have conducted a certain amount of research and achieved certain results in [4]. However, due to the inherent system limitations of the quality evaluation, the existing studies are in a bottleneck and the algorithm performance improves slowly. As mentioned above, the development of visual perception theory provides a new development impetus for stereo video quality evaluation research, driven by a visual perception model, and has become the new research key.

The application scenarios of three-dimensional video are rich and play an irreplaceable role in the industrial field. At present, the research on stereoscopic video quality assessment (SVQA) is gradually emerging, which has attracted the attention of many scholars [5]. Compared with plane video quality evaluation, the factors affecting stereo video quality are more complex, including depth information, binocular competition, binocular suppression and binocular suppression. In early studies, there were few public stereo video quality assessment databases, which had a certain impact on the study of objective evaluation algorithms. In recent years, according to different research purposes and needs, researchers have established a number of stereo video datasets to provide the basis for the objective evaluation of stereo video quality [3,6,7].

In this field, Yang et al. [8] used three processed differential video blocks as input to 3D convolutional neural networks (3D CNN), which could effectively capture local space–time features and describe global time information, and then established a scoring fusion strategy according to global time clues, so as to achieve an accurate evaluation of 3D videos. Imani et al. [9] improved the 3D CNN model by extracting features from three aspects, space, motion and depth, and then the three-dimensional features were connected together through the full connection layer to obtain the quality score of the stereo video. Feng et al. [10] proposed a multilayer binocular fusion convolutional neural network with three branches. Specifically, branch 1 was a multiscale cross-dimensional attention unit to capture key semantic information; branch 2 was a binocular fusion unit to adaptively fuse left and right video branches; and branch 3 was a parallax compensation unit including a reinforcement module to provide parallax feature, which resulted in a network with a high accuracy.

It can be seen from the above content that the research on stereo video quality assessment has developed rapidly in the past decade, and the algorithms have also developed from artificial feature extraction to automatic learning of the quality perception mapping relationship using deep learning models. However, the traditional machine-learning-driven method is still the focus of research, because of the huge capacity of stereo video and the huge consumption of processing time and computing resources.

We propose an NR-SVQA model in this paper, which extracts the time-varying characteristics of stereo video frames in the frequency domain while maintaining the original size of stereo frames. The significant contributions are organized as follows:

- Temporal modeling is the key to capture spatiotemporal distortion in video. Affected by camera motion, speed changes and other factors, video data have extremely complex dynamics in the time dimension. In order to effectively capture such diverse motion patterns, a temporal adaptive module (TAM) is proposed, which generates a video-specific kernel based on its own feature mapping. The TAM can learn and obtain short-term information from the local time window. This information is generated from the global view, and it pays more attention to long-term goals.
- The framework describes video frames from two parts: local short-term relation and global relation. This model can be flexibly embedded into any 2D CNN framework and can still use the pretrained backbone network parameters without significantly increasing the complexity of the model.
- Rich performance verification experiments are performed. From the results, the prediction of this model is in good agreement with the subjective quality. Moreover, compared with existing methods, the proposed method has a higher visual quality perception prediction accuracy in both symmetric and asymmetric distortion databases.

## 2. Related Works

According to the research route and theoretical basis of their algorithms, the existing stereo video objective quality evaluation models can be divided into two categories, one is the plane extension model, the other is the stereo knowledge model [7,11]. In the early stages, the expansion from image quality evaluation or video quality evaluation to stereo video was the plane expansion. In order to quantify the impact of compressed artifacts on stereo video quality, Hewage et al. [12] used the image quality evaluation algorithms PSNR, SSIM and video quality model (VQM) to predict the video quality scores of the left and right eyes, and then took the weighted average of the scores of the two videos to obtain the objective quality of the stereo video [13]. From the results, it can be concluded that the performance of VQM was much better than that of PSNR and SSIM. Meanwhile, Chen et al. [14] also extended PSNR and MS-SSIM to SVQA. From the experimental results, it could be found that MS-SSIM performed better than PSNR. Further, Wang et al. found that when the image quality assessment (IQA) method was directly used to predict the quality of asymmetric distortion stereo video, there was a serious systematic deviation. Therefore, the authors applied the binocular competitive incentive model to predict the systematic bias, and the proposed FR SVQA model performance improved significantly. Specifically, the authors used the variance of local space to create local energy maps, and the local energy ratio of left and right videos could effectively provide binocular competition information, thus establishing a weight strategy for the left and right videos and correcting for systematic bias. In this way, the model could effectively predict asymmetric video with mixed distortion [6]. Fang et al. proposed a binocular competitive weighting method, which was based on the spatial frequency and temporal motion of the primary visual cortex, and the performance of the SVQA model was improved. Specifically, in the first stage, the spatial distortion of the video was captured using the image quality evaluation method, and the temporal distortion was estimated by the motion difference between the source video and the distorted video. In the second stage, the structural strength (SS) and motion energy were obtained by the gradient and frame difference (ME). By simulating the binocular competition between SS and ME, a new weighting strategy was established and the spatiotemporal distortion estimation of the first stage was carried out, so as to obtain the objective score of the stereo video and correct the system deviation [15].

The stereo perceptual knowledge model does not rely on the existing image/video quality assessment methods, and focuses on constructing the stereo video quality assessment model directly. Jin et al. proposed a method based on block matching. Specifically, similar blocks in the left and right video frames were found and combined into 3D video blocks. When correcting PSNR, the mean squared error within the 3D discrete cosine change was used to measure the distorted video quality. Regarding the defects in accuracy and robustness of the planar extension method, Galkandage et al. [16] completed the evaluation of the accurate stereo video quality by measuring the quality using the extended binocular energy, which was based on two visual phenomena occurring in complex cells, namely binocular suppression and repetitive excitation. Appina et al. used the SSIM average between consecutive video frames to evaluate the overall motion of each video. The video motion and spatial quality were modeled, and the unsupervised image quality evaluation method was used to predict the spatial quality. Inspired by GGD, the author established the statistical dependence between the motion of the stereo video and the disparity subband coefficient of the space as a binary GGD (obeying a bivariate generalized Gaussian distribution, BGGD) model, and used the multivariate Gaussian to model (MVG) it [17]. Inspired by free energy principles and binocular vision mechanisms, Chen et al. proposed a depth video quality evaluation model closely related to stereo perception. Specifically, the model included two parts: autoregressive prediction-based disparity entropy (ARDE) and energy-weighted content measurement, in which the natural scene statistics of the two stereo channels were combined with ARDE to verify the sensitivity of video texture and frame difference to quality. In stereo video processing of video coding (HEVC), this method is widely used and performs well. Experiments have shown that the method still maintains good performance and in the case of other types of distortion [18]. Hou et al. used oriented local gravitational force (OLGF) statistics to extract local gravity responses from monocular maps, product images and frame difference maps and mapped the gravity response statistics to the quality score of stereo video [2] using SVR.

## 3. Method of SVQA

**Figure 1:**
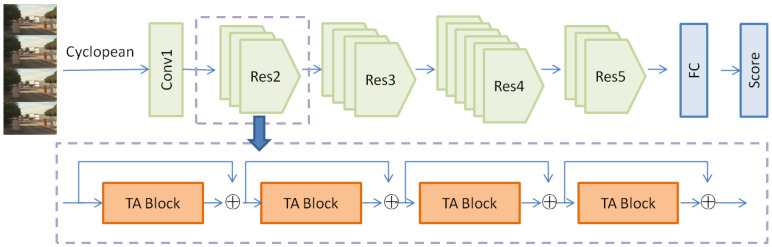
The overall framework of TAM.

According to the importance of spatiotemporal characteristics to stereo video, a time-adaptive stereo video quality assessment model is proposed. The algorithm framework is shown in Figure 1. The monocular image which conforms to the characteristics of human stereo perception was synthesized from the left and right video frames and input into the backbone network embedded in the time-adaptive model to perceive the stereo video quality.

### 3.1. Stereoscopic Formation

The perception of binocular competition is not independent of the complete stimulus intensity of each perspective but related to the relative stimulus intensity of both. When the weighting coefficient is positively correlated with the stimulus intensity, this can be explained by a model based on biology. In this process, the left and right image stimuli are measured by the local energy of the response of a group of Gabor filters. Binocular competition is a local multiscale phenomenon, so the method of broadening the horizontal model is a common method to simulate the synthesis of look-around images. The stereoscopic view synthesized as a look-around image is parallax-compensated, and the view image is illuminated to the spatial coordinate system of the left view image. Therefore, the linear model is used to synthesize the panoramic image, which is expressed as:

(1) $$C\left(i,j\right)=W_{L}\left(i,j\right)\times I_{L}\left(i,j\right)+W_{R}\left(\left(i+d\right),j\right)\times I_{R}\left(\left(i+d\right),j\right)$$

where *C* is the look-around image, which is the simulated image, $I_{L}$ is the image on the left and $I_{R}$ is the image on the right; *d* is the parallax index of the $I_{L}$ pixels corresponding to $I_{R}$. The $W_{L}$ and $W_{R}$ weights are calculated from the amplitude response of the normalized Gabor filter:

(2) $$W_{L}\left(i,j\right)=\frac{G_{L}\left(i,j\right)}{G_{L}\left(i,j\right)+G_{R}\left(\left(i+d\right),j\right)}$$

(3) $$W_{R}\left(i+d,y\right)=\frac{G_{R}\left(i+d,j\right)}{G_{L}\left(i,j\right)+G_{R}\left(\left(i+d\right),j\right)}$$

where $G_{L}$ is the sum of the convolution responses of the left image to the Gabor filter, and $G_{R}$ is the sum of the right image. Due to the normalization of (6), when there is binocular competition, if the Gabor energy of the left stimulus increases, the right energy decreases, and vice versa.

### 3.2. Temporal Adaptive Module

**Figure 2:**
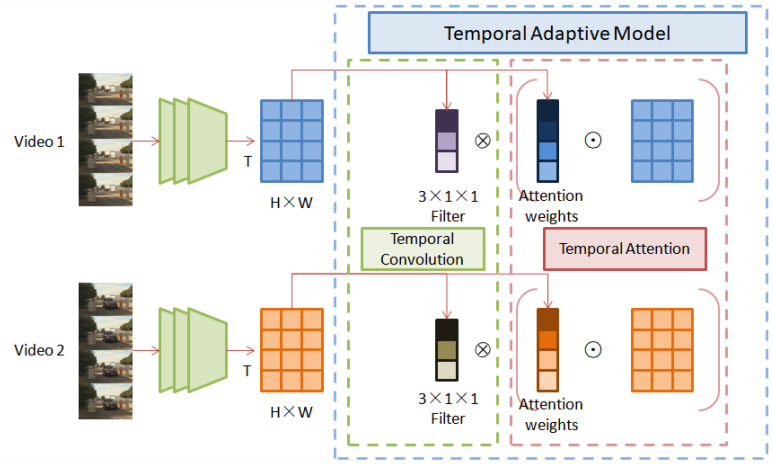
Two parts of temporal adaptive module.

Three-dimensional convolution is a generalization of 2D convolution. In 3D convolution, the 3D filter can be moved in all three directions (height, width, and channel) to output 3D data. At present, 3D convolution is widely used in the field of video understanding to extract temporal and spatial features in video. Although this simple expansion reflects a certain usefulness, it also lacks a comprehensive consideration of the temporal characteristics of video data, and the cost of computing is high. The video data show complex temporal dynamic properties such as camera motion and speed change. Different from the shared convolution kernel in 3D CNN, a time-adaptive module with a video-specific kernel is introduced to solve this problem. The TAM can generate a dynamic time core flexibly and effectively based on video features, so that the time information can be aggregated adaptively according to motion content [19]. The TAM can be easily embedded into an existing 2D CNN such as ResNet to generate a network architecture that can process video data. Figure 2 shows the process.

**Figure 3:**
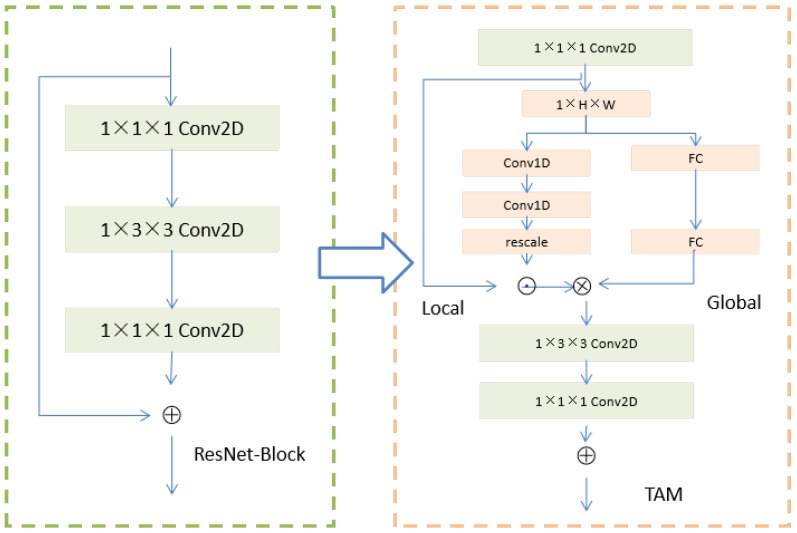
The concrete structure of the TAM module. On the left is an ordinary ResNet block and on the right is the TAM block with local and global feature description.

Specifically, for the feature map $X\in R^{C\times T\times H\times W}$, where *C* is the channel of features and *T* represents its temporal dimensions, *H* and *W* represent its spatial representation. A 2D convolution is used to capture spatial patterns, while the TAM is only used for temporal modeling. First, we use the global spatial average pool to compress the feature map:

(4) $${\hat{X}}_{c,t}=\phi {\left(X\right)}_{c,t}=\frac{1}{H\times W}\sum X_{c,t,j,i}$$

where *c* is the channel, *t* is the time, *j* is the height and *i* is the width index; $\hat{X}\in R^{C\times T}$ stands for aggregated spatial information. For convenience, ϕ is used to represent a function of aggregate spatial information, and the TAM is built based on 1D temporal features. The TAM is composed of a local part and global part, and Figure 3 shows the overall framework of the TAM, which enhances the salient features of the video by learning the position-sensitive feature graph; the position-invariant weights are generated and the time domain information is aggregated by convolution. The TAM is defined as:

(5) $$Y=G\left(\hat{X}\right)⊗\left(L\left(\hat{X}\right)⊙X\right)$$

where ⊗ and ⊙ are convolution operation and element multiplication, respectively. The output size of the global branch *G* is K×C and the output size of the local branch *L* is T×C×H×W. Both branches run on compressed feature image $\hat{X}$. It should be noted that the time information of the G and L parts is different. The L part uses time convolution to obtain short-term information, while the G branch leads the long-term time structure to the adaptive time aggregation full connection layer.

The short-term characteristics of video vary with time, so it is necessary to obtain a position sensitivity image to describe the local time structure. Specifically, local branches are constructed by using ReLU nonlinear time-convolution layer sequences:

(6) $$S=L\left(\hat{X}\right)=S_{i}\left(Conv1D\left(\delta \left(Conv1D\left(\hat{X},K,\frac{C}{\beta }\right),1,C\right)\right)\right)$$

where *S* is an important mapping, $S_{i}$ means the sigmoid function, *C* is the number of channels of the input tensor and δ is the ReLU function. Conv1D represents a temporal convolution, parameterized by the input, kernel size and the number of output channels. Since local branches are used to capture short-term goals, the size of kernel *K* is set to 3 and importance mapping is learned based only on local time windows. In order to speed up the convergence of the network, BN is applied after the first Conv1D, thereby reducing the channel from *C* to $\frac{C}{\beta }$. Then, the following Conv1D is followed by the sigmoid activation to generate the weight $S\in R^{C\times T}$. In order to match the size of *X*, we readjust *S* to $\hat{S}\in R^{C\times T\times H\times W}$ by copying in the spatial dimension:

(7) $$\hat{S}\in R^{C\times T\times H\times W}=S\in R^{C\times T}$$

where *c* is the channel, *t* is the time, *j* is the height and *i* is the width. The time-incentive mode is expressed as:

(8) $$Z=\hat{S}⊙X=L\left(\hat{X}\right)⊙X$$

where $Z\in R^{C\times T\times H\times W}$ is the activation graph and ⊙ represents element multiplication.

The focus of global branching is to generate an adaptive kernel that combines global context information into the TAM based on long-term relationships and captures aggregated location-sharing weights. A dynamic kernel is generated for each video frame in the global branch and time information is aggregated by convolution. In order to generate dynamic kernel efficiently, an adaptive kernel is learned at the channel level. The adaptive kernel that expects model learning only considers time-relation modeling and ignores channel correlation. Therefore, while maintaining the number of input channels, the adaptive kernel learned by TAM convolves the input feature map in a channel-level manner. The learning adaptive kernel for a specific channel is as follows:

(9) $$\theta_{c}=G{\left(\hat{S}\right)}_{c}=softmax\left(F\left(W_{2},\delta \left(F\left(W_{1},{\hat{S}}_{c}\right)\right)\right)\right)$$

where $\theta_{c}\in R^{K}$ is the adaptive kernel of channel *c* and *F* is a full connection (fc). Similar to the local part, the global part learns the adaptive kernel based on the compressed feature map ${\hat{S}}_{c}\in R^{T}$. However, unlike the local branch, the global part uses long-term information through the full connection layer to make the adaptive kernel learn. The two fc layers are stacked to improve the modeling capability of the global branches, and the positive aggregation weights are normalized from the learned cores using softmax functions. The learned aggregation weight $\theta =\{\theta_{1},\theta_{2},\cdots ,\theta_{C}\}$ is deployed in a convolution mode to obtain the time interaction between features.

### 3.3. Objective Quality Score Estimation

In the quality assessment task, we adopted ResNet50 as the backbone and added the TAM module. The TAM was embedded in the first Conv2D of the ResNet block. This embedding method did not change the topology of the network excessively, so that the ResNet block could be converted into a TA block efficiently and conveniently, and the weight of the ResNet block could be reused. The *T* frame was sampled as input, and the score of the *T* frame after fc was generated through average pool aggregation and a clip-level score. Time downsampling was not performed before the fc layer. It is worth noting that the trunk network had fewer restrictions on the insertion position and the number of TA blocks. Suppose the input nodes are $X_{1}$, $X_{2}$… $X_{n}$, the final quality score was expressed as:

(10) $$S=W_{1}*X_{1}+W_{2}*X_{2}+\ldots W_{n}*X_{n}+b$$

where *S* is the quality score, *W* is the weight coefficient, *n* is the number of nodes and *b* is the offset coefficient.

## 4. Experiments

In this section, a large number of validation experiments are reported to demonstrate the excellent performance of the proposed method. The four databases involved in the experiment and several indexes used to measure the prediction performance are introduced in detail. A comparison with existing methods on the whole database is provided. Finally, the performance of this method on different distortion types is verified, and the effectiveness of each module of the model is proved.

### 4.1. Databases and Indicators

The experiment was carried out on some public international stereo video databases, including the QI-SVQA database [20], NAMA-3DS1-COSPAD1 database [21], WaterlooIVC 3D Video Phase I database [22] and SIAT depth quality database [23]. The QI-SVQA database contains 9 original stereo videos in YUV 4:2:0 format with 25 fps and 450 corresponding asymmetric distortion samples, of which the number of H.264 distorted videos and Gaussian blur distorted video with multiresolution is 255. The distortion samples in the NAMA3DS1-COSPAD1 database were generated by encoding 10 original stereo videos with different scenes and distortion degrees, with a total of 100 samples with 25 fps and a resolution of 1920×1080. The types of distortion include H.264/AVC, JPEG2000, reduced resolution, sharpening and downsampling with sharpening. The WaterlooIVC 3D Video Phase I database has 4 undistorted stereo videos in YUV 4:2:0 format and 176 distortion samples with a 1024×768 resolution, using the HEVC encoder to pair the videos, which was quantified with QP={25,35,40,45,50}. In addition, the views QP={35,40,45,50} were processed by four degrees (σ={0,3.5,7.5,11.5}) of Gaussian low-pass filters. It contains symmetric distortion and asymmetric distortion samples. The SIAT depth quality database is a supplementary database of NAMA3DS1-COSPAD1, which provides depth quality scores. The database has a total of 160 distorted stereo videos, including 10 reference videos, 90 symmetrical distorted videos and 70 asymmetrical distorted videos. The distortion type is the same as that of the NAMA3DS1-COSPAD1 database. Each video in the dataset has its corresponding subjective score. The subjective scores of each dataset were scored by volunteers under strict rules [24].

The Pearson linear correlation coefficient (PLCC), Spearman rank correlation coefficient (SROCC) and root-mean-square error (RMSE) were used to measure the relationship between the objective prediction results and subjective evaluation scores, and then to verify the effectiveness of this method. The prediction accuracy and monotonicity of the prediction sample [25] were measured by the PLCC and SROCC, respectively. Higher values indicated a better performance of this method. The RMSE indicated the consistency of prediction, and [26] was referenced. Compared with the PLCC and SROCC, a smaller RMSE indicated a high performance. Before training the network model, 60% of the samples in the database were randomly selected to be used as the training set, 20% of the samples were used as the verification set, and the remaining 20% of nonoverlapping samples were used as the test set. To ensure the effectiveness of the algorithm model and eliminate the impact of individual differences on the overall performance, we repeated the algorithm 50 times, resegmented the data set each time, and finally took the average of the results of the 50 repeats as the final algorithm performance index. It is worth noting that standardized quality scores were used in training and testing. This section describes the experimental setup and implementation details in detail. The hardware used to perform the experiment was mainly based on an Intel (R) Xeon (R) CPU e5-2620 V4 and an NVIDIA GTX Titan XP GPU. Our proposed algorithm was based on the Pytorch deep learning framework. The network used the Adam optimizer, and the initial learning rate of the network was $10^{3}$. The mean squared error (MSE) was used to quantify losses.

### 4.2. Overall Performance

First, in order to prove the progressiveness of our method, we compared six models on the QI-SVQA database. PSNR, SSIM [27], MS-SSIM [28] and BRISQUE [29] are 2D models. Their principle is to process each frame of a stereo video, and then use the weighted average value of a single frame’s quality score to get the final prediction. SJND-SVA [30] and BSVQE [31] were implemented based on 3D-SVQA. Table 1 shows the results of the experiment. It can be seen from the table that the four quality evaluation indexes of BSVQE and the proposed method were good, indicating that they had a good prediction ability. In addition, for asymmetric distorted stereo videos, due to the different degree of distortion of the stereo pairs, the prediction results obtained by extending the 2D quality prediction model to the 3D model in the form of a weighted average of video frame quality scores had a certain systematic deviation. It could be proved that the performance of the model on an asymmetric distortion database was worse than that on a symmetric distortion database. Combined with the experimental results on the NAMA3DS1-COSPAD1 database to be introduced next, it can be seen that the sample size of the QI-SVQA database was 4.5 times that of the NAMA3DS1-COSPAD1 database. A large sample size is conducive to fully training the prediction model and obtaining relatively accurate prediction results. Therefore, the experimental results on the QI-SVQA database were better than those on the NAMA3DS1-COSPAD1 database. In addition, we also conducted performance comparison experiments between the SIAT depth quality database and various methods, and the results are shown in Table 2. Compared with other methods, our method showed a better performance in all aspects.

To further prove that this method was also applicable and performed well in stereo video quality prediction, we carried out performance comparison experiments on three databases. In particular, on the NAMA3DS1-COSPAD1 database, nine most advanced methods including six FR methods and three NR methods were used. DeMo_3D(MS-SSIM) [32], StSD [33] and MNSVQM [34] were implemented based on 3D-SVQA. Table 3 and Table 4 show the relevant experimental results. From the overall performance of these algorithms, it can be concluded that the proposed method was the most advanced algorithm. BSVQE based on binocular theory, and $DeMo_{3D}$(MS-SSIM) based on spatiotemporal characteristics also had a strong competitiveness. The results showed that the results of three-dimensional models were generally better than those of two-dimensional models, which indicated that the binocular phenomenon and stereoscopic parallax had a greater impact on quality perception.

We compared seven methods on the WaterlooIVC 3D video phase I database, and the results are displayed in Table 5. It can be seen that although the results of the proposed method and BSVQE were good, compared with the first two databases, the experimental results of each method on the database generally declined. We infer that symmetric distortion and asymmetric distortion were included in the WaterlooIVC 3D video phase I database. The composition of this database was more complex than other databases, so the quality prediction was more difficult. In addition, some algorithm codes used for comparison were not public. Not all algorithms could be implemented on all databases. Therefore, the comparison methods on each database were slightly different.

### 4.3. Experiments under Different Distortion Types

To comprehensively analyze the sensitivity of the proposed method to the quality degradation of different distortion types in stereo video, we designed experiments to test the prediction performance of different distortion types. In brief, 60% of a single distorted sample was used for training, 20% for the verification set and the remaining 20% for testing, which was the same as for the whole database. The relevant results are displayed in Table 3 and Table 1. From the results, it can be seen that compared with the existing comparison methods, the proposed method had a strong ability to deal with H.264 distortion. Meanwhile, BSVQE, BRISQUE and $DeMo_{3D}$(MS-SSIM) had a significant prediction ability for H.264 distortion. In addition, we also observed that the FR method had a good evaluation performance for the distortion of JPEG2000. In addition to the proposed method, BSVQE also showed a good ability to identify the degree of fuzzy distortion. Since the WaterlooIVC 3D video phase I database only contained HEVC distortion types, there was no need to analyze the HEVC distortion types.

### 4.4. Ablation Experiment

It is important to explore the overall functionality of the proposed approach, and the performance of each of its components is valuable. Since the impact of the TAM structure and the local and global parts of the TAM on the video quality perception of the model is unknown, in order to verify the sensitivity of the TAM model and TAM components to stereoscopic video quality, ablation experiments were designed, and the experimental results are shown in Table 6. “Ours w/o TAM”, “Ours w/o global” and “Ours w/o local” strategies were designed. By comparing the above three groups of strategies, it can be concluded that the TAM model and its components played an important role in improving the algorithm performance. At the same time, since the performance of “Ours w/o TAM” was the lowest, it can be shown that the two parts of the TAM did not interfere with each other. In addition, the experimental results showed that the time-domain characteristics were highly sensitive to the quality of the stereo video, which was suitable for quality assessment tasks.

## 5. Conclusions

This paper presented an objective evaluation method of stereo video quality based on the time domain. In view of the difficulty in obtaining video time-domain features and the low computational efficiency of models specially proposed by 3D CNNs, this paper introduced a time-adaptive model in the field of video quality evaluation for the first time, to establish the correlation between stereo video frames, so as to realize the time-domain connection between frames. The model was composed of local and global parts and could extract the time-domain characteristics of video comprehensively. In addition, the model could be easily embedded into an existing 2D CNN backbone network without significantly increasing the network parameters. From the experimental results on the NAMA3DS1-COSPAD1 database, WaterlooIVC 3D video phase I database, QI-SVQA database and SIAT database, the prediction results of this model were very close and similar to the subjective evaluation quality score. Furthermore, we will develop representative sequences in the future and further tap its potential.

## Figures and Tables

**Table 1:** Performance comparison of various methods on the QI-SVQA database. The best results are in bold.

Category		FR				NR		
	**Method**	**PSNR**	**SSIM**	**MS-SSIM**	**SJND-SVA**	**BRISQUE**	**BSVQE**	**Ours**
H.264	PLCC	0.6595	0.8371	0.8401	-	0.8704	0.9371	**0.9450**
	SROCC	0.8437	0.8566	0.8546	-	0.8446	**0.9379**	0.9334
	RMSE	0.74381	0.5413	0.5368	-	0.4791	-	**0.3133**
Blur	PLCC	0.6933	0.8342	0.8576	-	0.8493	0.9568	**0.9666**
	SROCC	0.8417	0.8420	0.8607	-	0.8306	0.9505	**0.9563**
	RMSE	0.7186	0.5498	0.5129	-	0.5202	-	**0.2483**
Overall	PLCC	0.7223	0.8346	0.8472	0.8415	0.8525	0.9394	**0.9520**
	SROCC	0.8361	0.8476	0.5567	0.8379	0.8448	0.9387	**0.9458**
	RMSE	0.6878	0.5478	0.5284	0.5372	0.5210	-	**0.2994**

**Table 2:** Performance comparison of various methods on the SIAT database. The best results are in bold.

Category		NR				
	**Method**	**PSNR**	**SSIM**	**DPDI**	**BSVQE**	**Ours**
H.264	PLCC	0.7043	0.6932	0.6862	0.8898	**0.8914**
	SROCC	0.6228	0.6656	0.5982	0.8225	**0.8310**
	RMSE	0.5721	0.5807	0.5659	0.3537	**0.3523**
JPEG2000	PLCC	0.3900	0.5018	0.4311	0.6523	**0.7611**
	SROCC	0.3156	0.2141	0.3196	0.5503	**0.6611**
	RMSE	0.3789	0.3559	0.3467	0.2888	**0.2626**
Overall	PLCC	0.6414	0.6097	0.5660	0.8810	**0.8862**
	SROCC	0.5197	0.5146	0.4858	0.8208	**0.8271**
	RMSE	0.5145	0.5315	0.5355	0.3074	**0.3051**

**Table 3:** Performance comparison of FR methods on QI-SVQA database. The best results are in bold.

Category		FR						
	**Method**	**PSNR**	**SSIM**	**MS-SSIM**	**SJND-SVA**	**StSD**	**DeMo_3D** **(MS-SSIM)**	**Ours**
H.264	PLCC	0.5758	0.7365	0.7885	0.5834	0.8020	0.9161	**0.9541**
	SROCC	0.5425	0.7172	0.6673	0.6810	0.7575	0.9009	**0.9441**
	RMSE	0.9463	0.7953	0.6955	0.6672	-	0.4654	**0.2523**
JPEG2000	PLCC	0.8073	0.9290	0.9439	0.8062	0.8433	0.9505	**0.9666**
	SROCC	0.7651	0.8879	0.9299	0.6901	0.8494	0.9326	**0.9611**
	RMSE	0.7362	0.4611	0.4327	0.8629	-	0.4074	**0.1426**
Overall	PLCC	0.6667	0.7981	0.8506	0.6503	0.7978	0.9242	**0.9562**
	SROCC	0.6230	0.7565	0.8534	0.6229	0.8162	0.9187	**0.9471**
	RMSE	0.8809	0.7121	0.5512	0.8629	-	0.4651	**0.3151**

**Table 4:** Performance comparison of NR methods on QI-SVQA database. The best results are in bold.

Category		NR			
	**Method**	**MNSVQM**	**BRISQUE**	**BSVQE**	**Ours**
H.264	PLCC	0.8850	0.9329	0.9168	**0.9541**
	SROCC	0.7714	0.8697	0.8857	**0.9441**
	RMSE	0.4675	0.3722	-	**0.2523**
JPEG2000	PLCC	**0.9706**	0.9055	0.8953	0.9666
	SROCC	0.8982	0.8503	0.8383	**0.9611**
	RMSE	0.2769	0.4904	-	**0.1426**
Overall	PLCC	0.8611	0.8897	0.9239	**0.9562**
	SROCC	0.8394	0.8490	0.9086	**0.9471**
	RMSE	0.5634	0.5236	-	**0.3151**

**Table 5:** Eight methods for overall and individual distortion performance on WaterlooIVC 3D Video Phase I. The best results are in bold.

		WaterlooIVC 3D Video Phase I		
**Category**	**Method**	**PLCC**	**SROCC**	**RMSE**
FR	PSNR	0.7085	0.5336	15.4507
	SSIM	0.3964	0.2872	20.1010
	MS-SSIM	0.4072	0.2969	19.9978
	StSD	0.7880	0.7543	-
	$DeMo_{3D}$(MS-SSIM)	0.8943	0.8806	9.4853
NR	BRISQUE	0.8711	0.8416	10.3788
	BSVQE	0.9343	0.8883	7.7882
	Ours	**0.9347**	**0.9027**	**7.4165**

**Table 6:** Ablation experiments on three databases.

	Component	Ours w/o TAM	Ours w/o Global	Ours w/o Local	Ours
NAMA3DS1-COSPAD1	PLCC	0.8765	0.9046	0.9482	0.9562
	SROCC	0.8481	0.8807	0.9213	0.9471
	RMSE	0.4908	0.4343	0.3252	0.3171
QI-SVQA	PLCC	0.9180	0.9265	0.9392	0.9520
	SROCC	0.90251	0.9177	0.9311	0.9458
	RMSE	0.3549	0.3678	0.3366	0.2994
WaterlooIVC Phase I	PLCC	0.9051	0.9198	0.9159	0.9347
	SROCC	0.8732	0.8891	0.8870	0.9027
	RMSE	8.8049	8.2847	8.4354	7.4165

## Data Availability

Not applicable.
